# Steering coherence in quantum dots by carriers injection via tunneling

**DOI:** 10.1515/nanoph-2022-0184

**Published:** 2022-06-21

**Authors:** Igor Khanonkin, Sven Bauer, Ori Eyal, Johann Peter Reithmaier, Gadi Eisenstein

**Affiliations:** Technion – Israel Institute of Technology, Andrew and Erna Viterbi Faculty of Electrical and Computer Engineering and Russel Berrie Nanotechnology Institute, Haifa, Israel; University of Kassel, Technische Physik, Institute of Nanostructure Technologies and Analytics, Center of Interdisciplinary Nanostructure Science and Technology (CINSaT), Kassel, Germany

**Keywords:** coherent control, quantum dot, tunneling injection

## Abstract

Coherent control is a key experimental technique for quantum optics and quantum information processing. We demonstrate a new degree of freedom in coherent control of semiconductor quantum dot (QD) ensembles operating at room temperature using the tunneling injection (TI) processes in which charge carriers tunnel directly from a quantum well reservoir to QD confined states. The TI scheme was originally proposed and implemented to improve QD lasers and optical amplifiers, by providing a direct injection path of cold carriers thereby eliminating the hot carrier injection problem which enhances gain nonlinearity. The impact of the TI processes on the coherent time of the QDs was never considered, however. We show here that since the cold carriers that tunnel to the oscillating QD state are incoherent, the rate of injection determines the coherent time of the QDs thereby controlling coherent light–matter interactions. Coherent interactions by means of Rabi oscillations were demonstrated in absorption and for weak excitation pulses in the gain regime. However, Rabi oscillations are totally diminished under strong excitation pulses which increase the rate of stimulated emission, causing the tunneling processes to dominate what shortens the coherence time significantly. Since the tunneling rate, and hence, the coherence time, were controlled by the optical excitation and electrical bias, our finding paves the way for TI-based coherence switching on a sub-picosecond time scale in room-temperature semiconductor nanometric structures.

## 1 Introduction

Various coherent interactions in room temperature active quantum dot (QD) ensembles have been demonstrated in the past few years [1] by employing pulse excitations with durations shorter than the coherence time which is usually of the order of 1 ps: starting with Rabi oscillations including coherently controlling them, Ramsey fringes, photon echoes and most recently coherent revival in a QD amplifier [2], in which high quality InAs/InP QDs [3] enable an exceptionally long room temperature dephasing time, longer than 5 ps. The coherence and dephasing times are determined by carrier–phonon scattering (and hence depends on temperature) as well as by carrier–carrier scattering which depends, in turn, on the injected drive current. Observation of Rabi oscillations was also reported in room temperature active QD amplifier based on InAs/GaAs [4] and predicted to induce self-mode locking in InAs/InGaAs QD ring lasers [5, 6].

Semiconductor laser structures based on tunneling injection (TI) were introduced in the 1990s as a mean to reduce threshold currents and temperature sensitivities as well as improving modulation bandwidths [7–9]. TI relies on a reservoir of cold carriers in the form of a quantum well (QW), which is separated by a thin energy barrier from the laser active region. Under high stimulated emission rates, cold carriers tunnel efficiently to the active region. This diminishes the detrimental role of hot carriers that relax from high energy states so that the gain non-linearity is reduced [10] and the laser performance improves.

The TI process itself was studied theoretically [11–16] and experimentally [7, 9, 16], [17], [18], [19], [20], [21], [22], [23] in both QW and QD structures. A detailed investigation of the TI mechanism in a TI-QD amplifier was presented in [16], where two simultaneous processes, resonant and non-resonant TI were identified using multi-wavelength pump probe measurements, with their efficiency shown to depend on the level of carrier injection.

TI-QD lasers have been investigated extensively in the context of improving laser performance. What was never considered and is addressed here is the impact of the high rate of tunneled carriers on the coherent properties of QDs. Furthermore, no scheme to harness the TI processes to control the coherence properties of QDs has been proposed.

In this article, we propose and demonstrate a new type of coherent control in a QD ensemble. We show that in a TI-QD amplifier, the excitation pulse energy determines the rate of stimulated emission and correspondingly the rate of carrier injection by TI. Since the tunneled carriers are incoherent, they curtail, in turn, the coherence time of a room temperature QD ensemble, thereby enabling or diminishing the observation of coherent light–matter interactions on sub-picosecond time scale.

Control over the coherence time is determined via the observations of Rabi oscillations that are imprinted onto the temporal pulse profiles due to the coherent interaction with the QDs. In the gain regime, the pulse amplitude profile shows no input power dependence. In the more sensitive instantaneous frequency profile however, a low input pulse energy yields clear Rabi oscillations which diminish for high input powers. High excitation powers lead to a high rate of tunneled carriers that shorten the QD coherence time to a degree that Rabi oscillations are no longer possible.

An exact quantitative determination of the coherence time [24, 25] is not possible from a single pulse experiment, which exhibits two Rabi flops at most (as described below). Nevertheless, the single pulse experiment provides an upper bound for the coherence time, that was found to be 250 fs. The exact coherence time can be extracted in a two pulse Ramsey experiment [26]. In absorption, the pulse amplitude profile exhibits self-induced transparency [1] and the instantaneous frequency always has an oscillatory nature since no tunneling takes place.

## 2 Experimental realization

**Figure 1:**
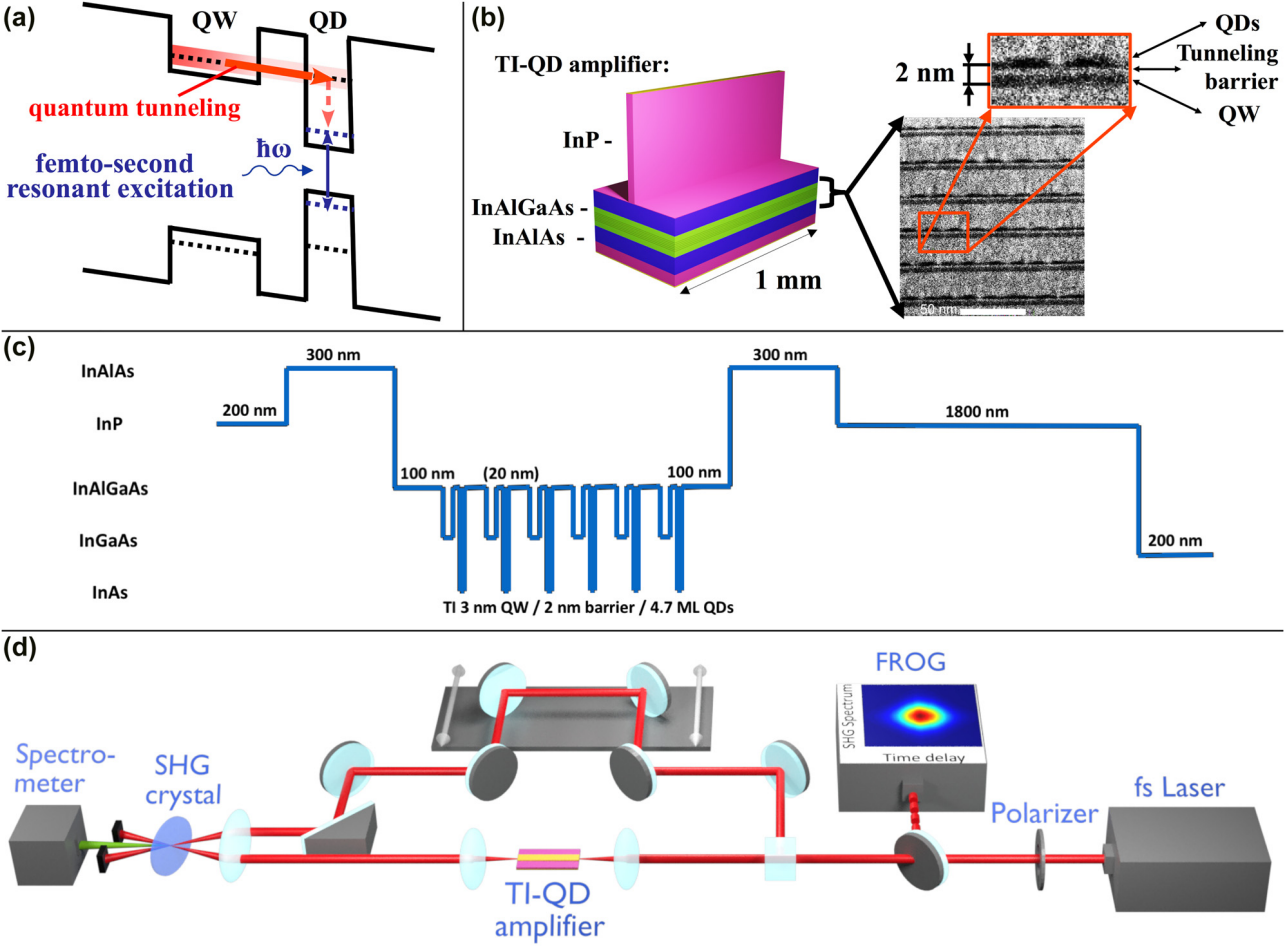
Coherent light-matter interaction in room temperature TI-QD amplifiers. (a) Schematic description of resonant tunneling and the coherent light–matter interaction between QDs and a resonant optical excitation. (b) A TI-QD amplifier comprising a ridge waveguide structure. The inset shows a high-resolution transmission electron microscope cross section of the six QDs–QW pairs of layers with the barriers separating them. (c) Energy band diagram of the laser structure comprising six layers of QD–QW pairs. (d) Schematic of the experimental XFROG system.

Figure 1a shows schematically the process of resonant tunneling in a TI-QD structure, mediated by energy level hybridization [14] in the presence of a large forward bias that bends the energy bands significantly. The experiments made use of a TI-QD amplifier designed for operation at 1550 nm. The TI-QD laser structure is depicted schematically in Figure 1b together with a cross section image of the epitaxial layers obtained by high resolution transmission electron microscopy. The TI-QD sample included six periods of 3-nm-thick In_0.532_Ga_0.468_ As injector QWs, separated from the InAs QD layers by 2 nm thick In_0.528_Al_0.238_Ga_0.234_ As barriers. The QDs were grown by molecular beam epitaxy in the Stranski–Krastanow mode with a high-density of 3 × 10^10^ cm^−2^. The energy band diagram is shown in Figure 1c. The amplifier was formed by a 2 μm wide, 1 mm long ridge waveguide whose end facets were anti-reflected coated. More details of the epitaxy process and the device fabrication are given in [16].

Cross-correlation frequency resolved optical gating (XFROG) [27] was employed to characterize the temporal profile of the pulses emerging from the amplifier. The experimental system is shown in Figure 1d. A Toptica FemtoFiber Pro laser was employed for generating excitation pulses centered at 1.54 μm. The pulses were characterized by an FROG measurement and found to be nearly transform-limited with a duration of 90 fs. The pulses were split with one arm (the reference) passing through a motorized delay line with a resolution of less than 1 fs. The pulses which were modified during propagating in the TI-QD amplifier were recombined with the reference pulses in a non-linear second harmonic generating (SHG) crystal. The SHG signal was captured by a spectrometer for scanned temporal delays between the modified and reference pulses. The measured XFROG trace was used to retrieve temporal intensity and phase (or the instantaneous frequency) profiles using a phase retreaval algorithm.

For a sufficiently large input pulse power, and hence pulse area, the period of the Rabi oscillations can be shorter than the pulse duration. This causes a modulation of the occupation probabilities commonly known as Rabi flopping [1]. Different parts of the pulse experience conditions that alternate between gain and absorption, what leaves a clear imprint on the temporal pulse profile.

## 3 Results and discussion

**Figure 2:**
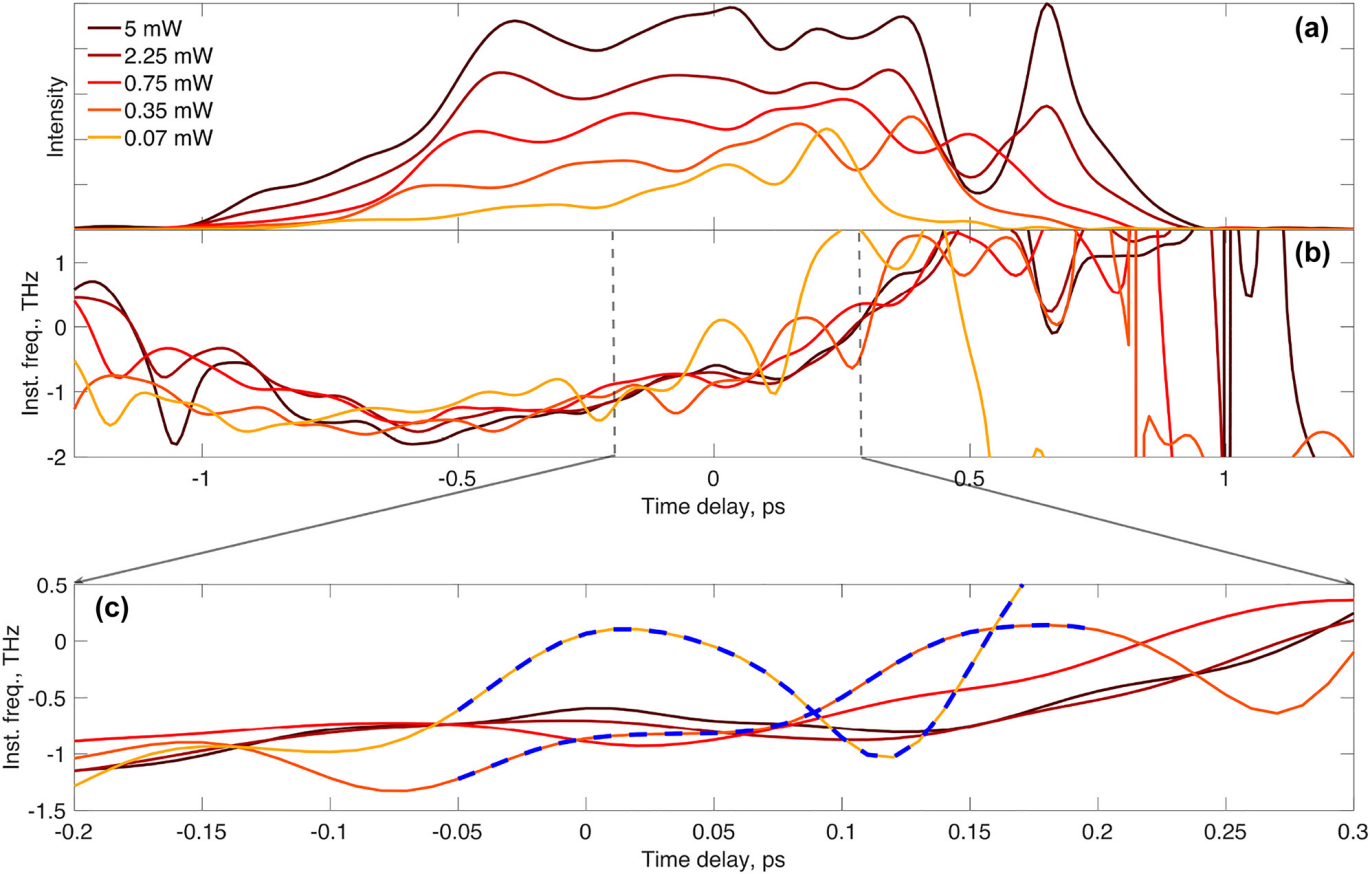
Experimental results of the temporal pulse profiles following the interaction with the TI-QDs for varies excitation pulse powers. (a) Normalized intensity profile, (b) and (c) instantaneous frequency profiles. The blue dashed line is shown for eye guidance. The TI-QD amplifier is biased to gain.

Figure 2 shows measured output profiles for a bias of 5 kA/cm^2^ which ensures the amplifier is in the gain regime and for several input powers. The pulse amplitudes are broadened and exhibit complex temporal profiles for all input powers. These result from group velocity dispersion and non-linear interactions of the gain region that mask coherent imprints on the pulse amplitude [28]. The evolution of the instantaneous frequency profiles originates from the plasma effect namely, the dependence of the refractive index on the excited carrier population. An increase in the rate of stimulated emission corresponds to a shortening of the oscillation period of the instantaneous frequency profile, in accordance with the increase of the Rabi frequency. In a standard QD amplifier (with no TI region) this increases the number of Rabi flops while shortening the time when the first flop occurs [1]. In a TI-QD amplifier, an increase in the rate of stimulated emission increases the rate at which incoherent, cold, carriers tunnel to replenish the occupation of the emitting energy level so that the coherence time shortens. The consequent result is shown in Figure 2 where the Rabi oscillations are observed on the instantaneous frequency profile only for low input powers of 70 and 350 μW. For those powers, the estimated coherence time is bounded to roughly 250 fs, which is sufficiently long to enable Rabi oscillations. However, for higher powers, those oscillations diminish since the high rate of tunneled carriers shortens the coherence time significantly. An enlarged image of the instantaneous frequency profile is shown in Figure 2c where the oscillations at low input power are clearly seen in the orange and red traces.

**Figure 3:**
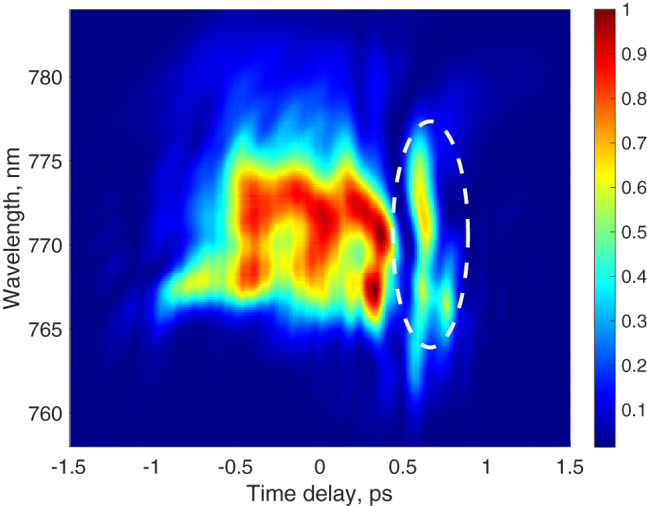
Spectrogram measured by XFROG of the pulse profile for a TI-QD biased to 5 kA/cm^2^ and for an excitation power of 6 mW.

The amplitude profile in Figure 2 reveals an extra peak at 0.75 ps. It originates from the excitation pulse which contains some residual energy outside the main pulse as is clearly seen in the XFROG spectrogram shown in Figure 3. It corresponds to one of the non-coherent light matter interaction phenomena discussed in [28]. In Figure 2c, the instantaneous frequency of the extra peak exhibits a minimum, which is in contrast to the oscillatory, hence coherent, nature of the profile during the main pulse. Moreover, the extra peak appears at a higher energy than the main pulse and is due to amplification of a side lobe of the imperfect input pulse.

**Figure 4:**
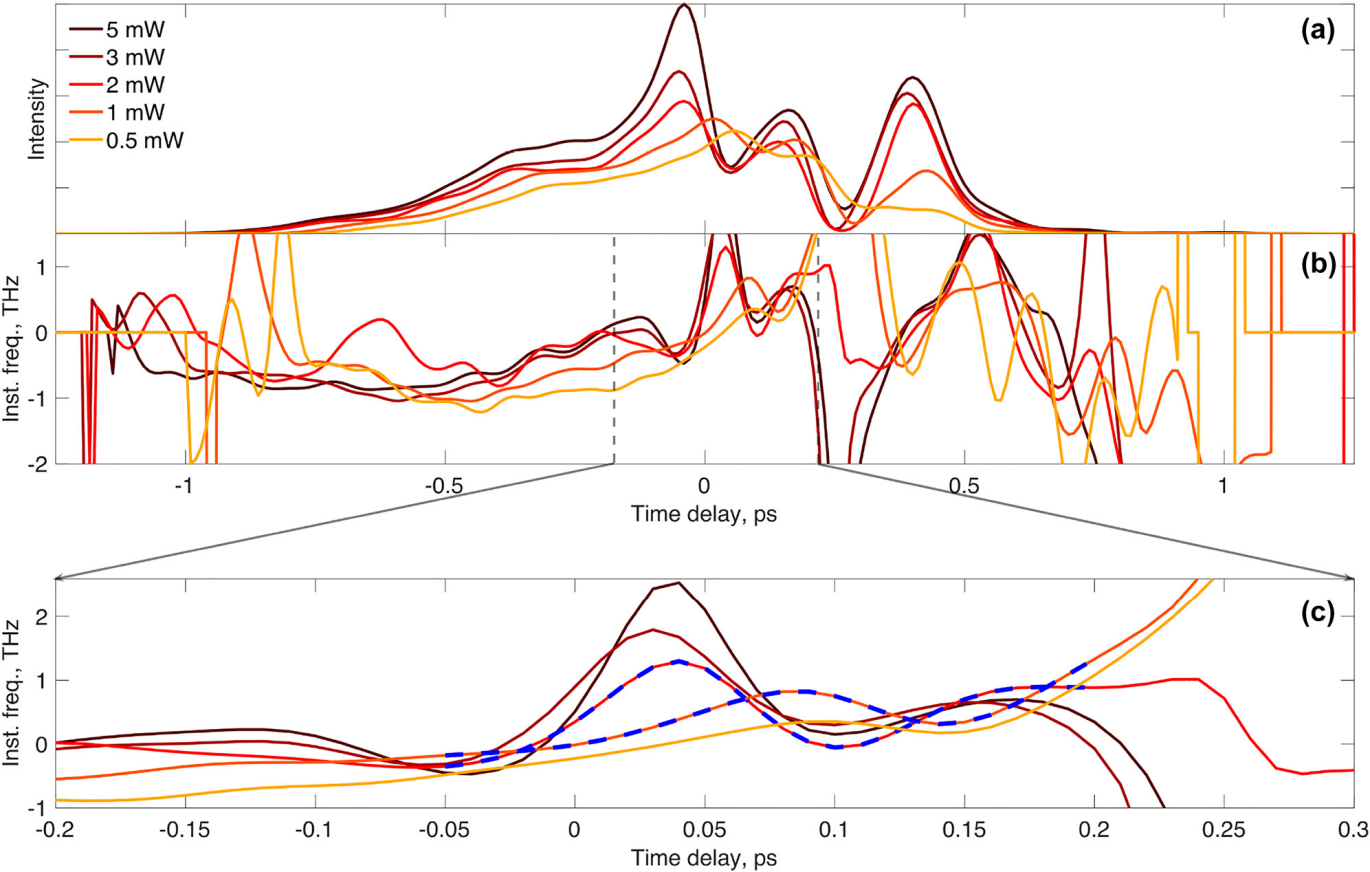
Experimental results of the temporal pulse profiles following interaction with TI-QDs for varies excitation pulse powers under absorption conditions. (a) Normalized intensity profile, (b) and (c) instantaneous frequency profiles. The blue dashed line is shown for eye guidance. The TI-QD amplifier is biased to absorption regime.

For a bias of 0.5 kA/cm^2^, the amplifier is in the absorption regime. Now the initial occupation probability of the electrons is higher in the valence band compared to the conduction band. This means that a complete flop of the populations transforms the medium into gain conditions. It causes pulse compression since its central portion experiences gain while the leading and trailing edges are absorbed, what is known as self-induced transparency [1]. A competing effect, two-photon absorption (TPA), affects mainly the peak of the pulse causing pulse broadening, which opposes the compression [1]. Nevertheless, the pulses do compress as the input power increases (see Figure 4). Moreover, they are clearly narrower than the ones under gain conditions proving that significant pulse shortening is caused by the state of the material.

The measurements conducted in the absorption regime yield the amplitude and instantaneous frequency profiles, depicted in Figure 4. The instantaneous frequency traces comprise two valleys which are clearly seen at high input pulse intensities. These signify two distinct amplification events occurring within the duration of the pulse and is a clear feature of the Rabi oscillation. The absorption profile exhibits also the independent extra peak (here at 0.5 ps). As in the gain regime, this peak plays no role in the coherent interaction we are studying. An enlarged image of the instantaneous frequency profile is presented in Figure 4c. The traces exhibit an oscillatory nature which is very clear at high input intensities but is also seen for weak pulses.

The fact that the instantaneous frequency, exhibits Rabi flops only for very low energy pulse excitation when the TI-QD amplifier is in gain and under absorption, sheds light on the ultra-fast relaxation processes mediated by tunneling. Though the fast relaxation process improves the dynamical performance of TI-QD laser, it simultaneously reduces the QD coherence time and hence hampers the occurrence of Rabi oscillations.

## 4 Conclusions

To conclude, we have demonstrated control over Rabi oscillations in a TI-QD amplifier. By modifying the rate of tunneling into the QD ground state via the amplifier bias and the excitation pulse energy, we control the coherence time and hence the occurrence of Rabi oscillations. Extracted profiles of the instantaneous frequency, exhibit a signature of Rabi flops for low excitation pulse energies in the gain regime and when the TI-QD amplifier is in absorption, Though, the fast tunneling process under a high rate of stimulated emission improves the dynamical performance of TI-QD lasers, it shortens the QD coherence time to a degree that Rabi oscillations are not possible. Controlling the input pulse intensity becomes a tool to tailor the coherence time in the gain regime, and enables to control Rabi oscillations and every other coherent light–matter interaction. For a low applied current, when the amplifier is in the absorption regime, the fast tunneling processes do not affect the electronic coherence in the QDs, and enables Rabi oscillations for all input powers.

In the present experiments, the rate of stimulated emission was controlled by the excitation intensity. An attractive alternative is to keep the excitation constant and change the bias to modify the stimulated emission rate thereby modifying the coherent time in a simple manner. However, a bias change modifies the gain and hence the dipole moment so that the control over the coherence is more complicated.
